# Conflicting Issues of Sustainable Consumption and Food Safety: Risky Consumer Behaviors in Reducing Food Waste and Plastic Packaging

**DOI:** 10.3390/foods11213520

**Published:** 2022-11-04

**Authors:** Gyula Kasza, Nina Veflen, Joachim Scholderer, Lars Münter, László Fekete, Eszter Zita Csenki, Annamária Dorkó, Dávid Szakos, Tekla Izsó

**Affiliations:** 1 National Food Chain Safety Office, Department of Risk Prevention and Education, 1024 Budapest, Hungary; 2 University of Veterinary Medicine Budapest, 1078 Budapest, Hungary; 3 Department of Consumer and Sensory Sciences, Nofima, NO-1431 Ås, Norway; 4 Department of Marketing, BI Norwegian Business School, NO-0484 Oslo, Norway; 5 Department of Informatics and Sustainability Research, University of Zurich, CH-8050 Zurich, Switzerland; 6 Rådet for Bedre Hygiejne, 2100 Copenhagen, Denmark; 7 Hungarian University of Agriculture and Life Sciences (MATE), 2100 Gödöllő, Hungary

**Keywords:** food-safety conflicts, risk communication, food waste, plastic packaging, consumer behavior, food-safety message

## Abstract

Food-related consumer decisions have an impact on the environment. However, trending patterns of sustainable consumption often pose a challenge for food-safety authorities: these initiatives may unintentionally compromise food safety. The objective of this review is to support public agencies in the integration of sustainability issues into food-safety risk communication schemes. Environmentally conscious but risky behaviors aimed at the reduction of food waste and plastic packaging were chosen for discussion and scrutinized based on expert opinions. Those expert opinions clearly indicated that a significant part of environmentally conscious behaviors, such as removing mold, eating expired perishable food, overstoring leftovers, avoiding single-use plastic packaging even when cross-contamination is a threat, and using reusable bags without cleaning for a long time, often contribute to food-safety risks. Short, easy-to-remember messages were collected for each recognized risky behavior; they concentrated on prevention or providing an alternative that was still environmentally sensible but kept food-safety risks low (such as planning ahead to avoid leftovers, freezing leftovers in time, and sanitizing reusable bags). The identified challenges and solutions might encourage authorities to rethink their risk-communication practices and integrate a sustainability aspect in them.

## 1. Introduction

Food-related consumer decisions, such as choosing what to eat, where to purchase, how much to consume, and what handling and disposal practices to use, clearly have an impact on the environment [1,2]. Sustainable consumption has recently become a dominant issue in consumer decisions in which both personal needs and social responsibility are considered [3]. Many factors contribute to consumption patterns; convenience, habits, value for money, health concerns, health risk perception, hedonism, and individual responses to social norms all have an impact to some extent [4]. Regarding food consumption, the effects of social norms are often more pronounced in food choices than the perceived risks of jeopardizing food safety, as socially “proper behavior” acts as an attenuation circumstance [5]. For example, people may be afraid to be judged for throwing out food, so they would rather consume an expired product if they think that would be more acceptable by their social environment [6]. The effect of social norms is significant because natural human desire is to belong to a community; therefore, people behave in order to comply with this ambition [7]. This desire is particularly strong in today’s younger generations (Millennials and those younger than them), which are also more receptive to meeting the criteria of sustainable consumption by being more concerned about the environment and more open to new trends in sustainability, such as zero-waste initiatives [8]. In contrast, older consumers may implement risky food-safety behaviors in favor of sustainability, not because of trends, but due to financial reasons [9,10]. Consequently, we might state that in light of the importance of sustainability issues nowadays, food safety—ensuring that food is not harmful and is appropriate for human consumption [11]—can be overlooked.

Food-safety regulations and guidelines issued by authorities are often regarded as too strict and contrasting to sustainability principles, though they primarily prioritize protection of human health [12]. For instance, officially ordered product recalls, withdrawals, and destruction of presumably hazardous batches often perceived as unnecessarily extreme measures and inspire various food-saving ideas. Other examples include confusion about food labeling and prohibition of selling food after expiration date in some member states [13]. Despite strict requirements and controls from food-safety authorities, more than 23 million people in Europe are affected by foodborne illnesses each year [14]; moreover, these types of diseases are tendentiously underreported in official records, due to several reasons [15]. According to public health statistics, more than 40% of foodborne illnesses were linked to households where practices originated due to sustainability reasons, contributing to the number of food-safety incidents [15,16]. The presence of the most common foodborne pathogens (e.g., *Bacillus cereus*, *Listeria monocytogenes*, *Streptococcus*, *Staphylococcus* spp., etc.) [17,18] can lead to problems for healthy consumers only if food-handling behavior promotes pathogen reproduction or does not inhibit pathogen growth (such as saving leftovers and ready-to-eat foods for too long or fostering cross-contamination through lack of packaging). Authorities are expected to react and adapt to changing market trends and consumer behavior in order to be able to keep up with emerging issues. By following and supporting sustainability-related initiatives, food-safety agencies will be able to identify and mitigate food-safety risks in time and also increase their credibility [19].

There are many contrasting issues in the field of food safety and sustainability, such as the role of local food systems and short supply chains, organic and other sustainable agricultural production methods, changing diets, development of novel foods, and better utilization of by-products and side-streams [20,21]. However, in this paper, the reduction of food waste and plastic food packaging were chosen for discussion, as they both represent emerging importance to consumers and are critical issues from a legislative aspect as well. The present paper reviews the most frequently observed food-safety issues in these two fields and attempts to resolve controversies. The research group (consisting of food safety and quality, food engineering, microbiology, sociology, risk communication, economics, and sustainability experts) also presents recommendations about the integration of sustainability issues into the food safety risk communication activities of public agencies.

## 2. Mitigation of Food Waste without Compromising Food Safety

Minimizing amount of waste and unnecessary usage of resources is among the principles of sustainable consumption [22]. Preventing food waste is essential, since food waste embodies uneaten food and all input used in its production (e.g., cropland, fertilizers and pesticides, water, feed of animals, energy, human resources, etc.) [23,24]. In addition to environmental impacts, ethical (e.g., fighting against hunger) and economic (e.g., production costs, household budget) aspects are not negligible [25].

Food waste arises in every step of the food chain; thus, mitigation should be a common goal, and responsibility is shared among food-chain actors [26]. On the consumers’ side, raising awareness and promoting good practices are especially important. EU countries should establish food-waste prevention programs according to the Directive (EU) 2018/851 [27]. In transposition of the Directive, several countries are implementing national waste-management plans and measures to reduce food waste. For instance, Bulgaria introduced the National Waste Management Plan 2021–2028 [28], the Greek Ministry of Environment set a strategic goal for the period 2021–2030 [29], Hungary aims to reduce food waste and losses through the National Waste Management Plan 2021–2027 [30], and Romania ordered the “Government Decision 92/2021” on its waste regime [31]. Incentives such as tax deductions for companies that save food by donation are also present in some EU countries [32]; this is an effective food-waste reduction policy that was introduced several years ago.

Changing habits about food waste is challenging. Consumers are usually not aware of their role in food-waste production, similarly to their awareness of their role in maintaining food safety [33]. In both areas, they often blame food-industry and hospitality units for foodborne illnesses and high amounts of food waste. However, the highest ratio of occurrence of diseases and proportion of wasted food is linked to household practices [34,35,36]. According to Skuland et al. (2020) [37], ignorance of the expiration date of perishable products, inadequate treatment of spoiled food, and irresponsible handling of leftover meals in order to avoid food waste are among the most common food-safety problems at a consumer level. As Watson and Meah (2012) identified [38], household budget, ethical issues and sustainability issues are often more important to consumers than minimizing food-safety risks.

A common problem is that people do not pay attention to the labeling of expiration dates either during shopping or at home, nor are they aware of different types of expiration dates [39]. More than half of consumers do not recognize the difference between use-by and best-before dates [40], resulting in approximately 10% of food waste [41]. In the case of perishable products, it is a common belief that use-by dates are overly cautious and therefore food can be consumed safely for 1–2 days after the expiration date [42]. Additionally, edibility of these products is frequently “probed” based on sensory characteristics, although the presence of pathogens (including viruses) and their toxins is usually not accompanied by changes in taste, smell, or texture of food [43]. Other “at-home tests” or traditions for checking the safety status of food products also failed when academics tried to validate them. For example, putting eggs into water to check freshness does not indicate the presence of *Salmonella* [44,45]. However, consumption of eggs after expiration date is a common and risky habit in some countries [45]. Long-shelf-life foods—foods with a best-before date—regularly end up in the trash, generating unnecessary food waste once the expiration date had passed by only a few days or weeks [46].

Regarding spoiled, moldy food, a false public belief is that removing visibly contaminated parts can save the product [47,48], a practice which is frequently observed when handling cheeses, bread, jams, fruits, and vegetables [37,49]. Possible reasons for this risky behavior stem from various negative emotions about discarding seemingly salvageable food [6]. Some people decide to use moldy products (even when a certain level of risk is recognized) under time pressure, for instance when being in the middle of cooking and the concerned ingredient is essentially needed.

Generally, consumers do not understand that invisible hyphae of molds are extended to the entire food. Several fungi species produce mycotoxins that cannot be detected without laboratory equipment. Moreover, cooking does not deactivate mycotoxins. Therefore, even if a consumer takes those extra steps to make spoiled food safer, the hazard will not be eliminated [49,50]. In a few special cases, some moldy products might still be possibly saved by cutting out contaminated parts (hard cheeses, firm vegetables such as carrots, and hard cured-meat products such as salami) [47,51]. However, communicating such detailed pieces of information to consumers (for instance, the exact types of foods that are suitable for saving, and how much should be removed from the product to ensure safety) is nearly impossible [52]. To keep it simple and safe, it is better to recommend moldy products to be discarded.

Handling leftover meals is important for food-waste prevention but has also proven to be an area of concern regarding food safety, according to Skuland et al. (2020) [37]. Moreover, meals prepared by consumers or their family members are bearing an emotional value, that influences food safety related decisions [6]. Eating leftovers after more than one cycle of storage and reheating or storing at too high of a temperature for too long are among observed questionable practices [37]. Storing food at higher temperatures facilitates microbiological growth; moreover, microbes will become more resistant to heat if food is repeatedly reheated at sublethal temperatures [53,54]. Spore-forming microbes will also germinate after heating and can grow if the temperature is over 4 °C [55]. An extreme but attention-grabbing precedent in inadequate treatment of leftovers is the case of a young adult in Brussels who died of food poisoning caused by *Bacillus cereus* toxins in poorly stored spaghetti [56].

Another excellent example of sustainability–safety controversy is when consumers focus on saving unavoidable food waste instead of preventing avoidable food waste. For instance, preparing “banana-peel bacon” is now a trending issue among consumers, even though fungicides and insecticides on the peels of bananas may pose food-safety risks even after frying, as most pesticides do not decompose at that temperature [57,58]. Bananas are thrown away in the most significant amount among fruits due to improper storage [59]. Instead of food-saving solutions that seem simple and quick but elevate level of risk, a focus should be placed on methods that prevent food waste and do not compromise food safety [15].

The main problem regarding all the cases mentioned above (ignorance of expiration dates, removing mold, poor leftover handling, saving banana peels instead of whole bananas) is how consumers attempt to solve sustainability challenges by performing risky practices.

### Risk-Communication Messages to Disband Conflicts between Ensuring Food Safety and Food-Waste Reduction

Consumer behavior and attitudes have to be moved toward a balance through effective communication strategies, which prioritize food safety, but aims food waste prevention as much as rationally possible. This endeavor includes research regarding how safety messages should be communicated by food-safety authorities and understood by consumers; how messages influence behavior, shame, hesitancy, or discomfort related to discarding food; and how to influence consumers’ responses [59]. A strong emphasis on educational communication (especially children’s education) [60,61], virtual demonstrations, and citizen engagement is necessary to allow people to voice their opinions and perceptions in a “safe space”. These communication activities should be based on genuine dialogs and address myths and misconceptions rather than picking out individual practices, to prevent the participants from being repressed, ignored, or ridiculed [62].

Regarding the messages to be communicated, short, clear, simple, and easy-to-remember advice should be formed, similarly to any other effective risk communication. In Table 1, a collection of advice focusing on food-waste prevention is listed and related to risky behaviors mentioned in the previous section. Food-safety messages, which might contradict principles of sustainable consumption are also presented for each consumer behavior.

Instead of telling consumers what not to do, it is better to present good practices that keep food waste low without increasing food-safety risks [69]. In most cases, prevention is the key: for instance, planning meals ahead (e.g., with a weekly menu considering personal preferences and portions) and knowing what to look for during shopping (e.g., expiration date, indications for storage and usage) [70]. Consumers should be aware of the FIFO (first in, first out) principle and keep track of their food storage (consuming products that expire sooner first) [71]. Instead of disposing of moldy parts of various foods, planning and proper storage should be emphasized (e.g., refrigerator temperature, freezing as a prevention method, where to store certain food categories) [72]. Guidance for handling of leftovers should concentrate on avoiding generation of leftovers in the first place, such as how to choose portions [73].

## 3. Role of Plastic Packaging in Food Safety and Sustainable Consumption

Packaging has a multifaceted role in the life cycle of food; it is a physical protective barrier [74] and a communication and marketing platform [75]. It also provides resistance to tampering as well as enabling convenient handling, transportation, and storage [76]. Although these aspects are all important, emphasis is constantly shifting in parallel to changes in legislation and consumer expectations [77], resulting in turbulent evolution during recent decades. Nowadays, expectations towards packaging have become rather complex. In addition to protection and information, sustainability aspects have also come into the light [78].

Although reducing the amount of packaging along the food chain is an unequivocal societal expectation, the function of food packaging in preserving food safety and quality is also unquestionable. Food manufacturers are obliged to comply with current European Union directives and take steps to reduce usage of lightweight plastic carrier bags and withdraw other single-use plastics [79,80]. Different countries use different approaches to implement the European Packaging Directive 94/62/EC [81] into national law. For example, in Croatia, there is a refund–fee system for managing single-use plastic packaging [82]. In France, the “Circular Economy Law” (Law No. 2020-105 of 10 February 2020) focuses on recycling channels [83]. The Portuguese government determined the obligation of non-use of single-use plastics for food [84] and prohibited use of ultralight plastics [85]. Similarly, the UK introduced a complex strategy that includes measures regarding taxes, separate waste-stream collection, deposits on bottles and cans, and stimulation of recycling [86]. However, regardless of the efforts and approaches used, aspects of food safety cannot be compromised at any step of the food chain due to global intention to reduce use of plastic packaging.

Consumers tend to judge packaging in an extremist way; in general, they overestimate the negative environmental aspects of food packaging but underestimate its role in food safety. According to consumers’ assumptions, more than half of the total carbon footprint of a food product is related to the packaging [87]. In fact, the actual data on the carbon footprint ratio of packaging compared to the total carbon footprint of the product is only 1/30 [88]. Consumers tend to consider disposable packaging an enemy, even though it significantly contributes to maintaining food safety and, due to longer shelf life, even facilitates a more sustainable food chain [89]. According to the literature, estimated food loss due to lack of proper packaging has a bigger negative effect on the environment than the positive effect of simplification or complete abandonment of packaging [90,91]. This principle is especially true for food categories with high environmental impact, such as meat products or dairy products [92].

Packaging is the most efficient physical barrier to protect food; unpacked food is prone to food-safety risks. Elimination of single-use packaging results in the spread of reusable packaging materials. Single-use plastic bags—used for bakery products, vegetables, and fruits—not only are convenient but also help to prevent cross-contamination by separating food products. Replacing single-use plastic bags with reusable shopping bags may deliver new types of risks; consumers are often not aware of their own responsibility in maintaining the hygiene of these items (bags, boxes). Non-adequate washing and sanitizing of these containers can lead to cross-contamination. Certain retailers also serve high-risk food products, such as cheese, meat, and cold cuts, to consumers who bring their own containers. The hygienic status of those containers is not guaranteed, so even a safe food can become contaminated by an improperly cleaned box. Bacteria can also be transmitted from boxes to deli-counter tools, surfaces and personnel. Customer-owned takeaway containers in restaurants evoke similar problems. Although they deliver sustainability benefits and represent a cheaper option for consumers (compared to the cost charged for a takeaway box), the hygienic status of home-washed reusable boxes poses significant food safety risk [93]. Guidelines and protocols for retailers and hospitality actors might seem to be too rigorous in certain circumstances [94], but their main objective is to ensure food safety. According to general food-law stipulations, such as in the case of the 178/2002/EC regulation [95], food-business operators bear the primary legal responsibility for the safety of their products and services. However, food-hygiene protocols should accommodate to changing trends. New, improved practices have to be developed with the support of the authorities. For instance, reusable boxes with deposits could be introduced, or restaurants could provide a serving space for user-owned containers in a separate area.

Proliferation of package-free stores has heralded a new horizon in commerce. Even though the protective function of packaging is absent in these cases, the risk of contamination from consumers is significantly higher. The protective role of plastic packaging has been even more appreciated after the COVID-19 pandemic [96,97]. A previous fieldwork study [37] pointed out that package-free bulk products raise consumer concerns about other shoppers coming into physical contact with unpacked food products. The fear of contaminant transmission from people to food can contribute to unsafe food-handling practices in the household, such as rinsing raw chicken [98,99].

Additionally, because packaging serves as the primary communication platform between food manufacturers and consumers, lack of packaging can easily imply a lack of risk-related information for consumers [100]. In the case of bulk products, bulk-food containers in the shop must be equipped with the food label required by legislation, or personnel of the shop should be able to provide information upon consumer request. However, all necessary food-safety information (e.g., expiration date, storage circumstances) [101] vanishes after the product fills the consumer’s own food container, resulting in a possible knowledge deficit before consumption. The deficiency in consumer knowledge can pose food-safety risks and trigger household food waste [102].

### Implementing Effective Risk Communication in the Field of Plastic-Packaging Usage

The topic of plastic packaging is a controversial issue nowadays, emerging as a very real challenge for both food-business operators and consumers due to novel EU legislation. EU-level and national communication campaigns about the food-safety risks of eliminating plastic packaging are needed to tackle sustainability–safety controversies. For the time being, food-safety risks of zero/low-waste movements are not as widely researched as the topic of household food waste [103,104]. However, the primary direction of risk communication can be set. As food safety cannot be compromised for sustainability purposes, the use of protective packages is sometimes inevitable during shopping and handling of food [105]. This principle has to be the basic message in packaging-related risk communication. In addition to offering alternative ways of eliminating plastic packaging in everyday life, authorities’ targeted risk communication should also focus on proper handling of reusable alternatives, such as consumers’ own food containers and bags. Awareness-raising activities should draw attention to consumers’ responsibility in proper cleaning procedures (frequency of washing, ideal washing temperature, detergents) of reusable linen, cotton, and plastic bags to prevent cross-contamination and mitigate microbiological risks [106].

In the table below (Table 2), the most frequent risky consumer behaviors connected to reusable packaging materials are collected, along with pieces of sensible food-safety advice that considers sustainability aspects. These balanced messages are recommended to be used in the communication activities of food-control authorities.

As presented in Table 2, proper handling of reusable bags, their regular sanitization and avoiding risky foods (e.g., unpackaged meat, chicken, dairy products, eggs) to be put into them, is essential to maintaining food safety. Concerning unlabeled foods, raising consumer awareness about traceability within the household (for instance, keeping track of the expiry date) is a key message to communicate, as well as providing good storage practices.

During consumer campaigns, it is important to note that most single-use packaging materials (e.g., PET, paper) can be recycled through selective waste collection and processing systems. Unfortunately, many food packaging materials cannot be easily recycled due to their multi-layer structure [105]. During recent decades, development of novel packaging materials based on by-products and biodegradable materials has become an important research field [109,110], that can alleviate this challenge. The novel packaging solutions increasingly indicate that total rejection of single-use plastic is not the only path for a more sustainable food packaging. Therefore, sustainable communication messages should focus on sharing knowledge and consumer engagement in proper recycling [111], rather than elimination of protective packaging. Use of simple, well-designed packages and their selective collection and recycling might help to find the balance between protecting consumers’ health and sustainability.

## 4. Conclusions

**Figure 1:**
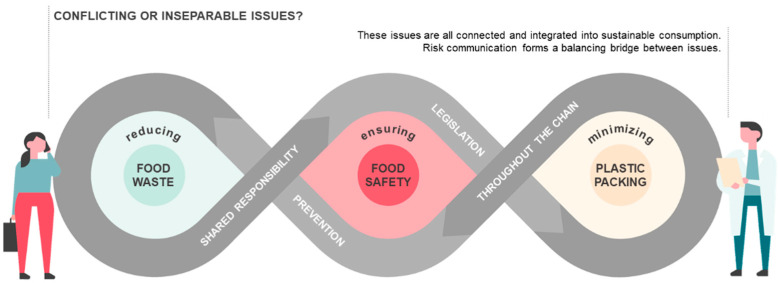
Connections between ensuring food safety, reducing food waste, and minimizing usage of plastic packaging (edited by the authors).

Surveillance of food safety along the food chain, with which sustainability issues have been entwined in recent years, is an unequivocal duty of food-safety authorities [112]. Reducing food waste and minimizing single-use plastic packaging are emerging issues, showing several similar characteristics (Figure 1). First of all, all actors along the food chain are responsible for handling these topics. The role of consumers is crucial in both fields, even when they are not aware of it. Although food safety and food-waste reduction can be assured by EU-level and national regulations in the pre-consumer stage (during agriculture, food processing, and retail), maintaining food safety and reducing food waste in the consumer stage is a far more problematic issue. Reduction of plastic packaging also raises challenges at the consumer level because misperceptions and bad practices can lead to food-safety risks in daily use of reusable packaging materials. Management of these complex areas (especially minimizing use of plastic) demands continuous research and innovation and requires communication across different industry sectors [113].

Awareness-raising activities and risk-communication campaigns are needed to address these issues without blaming consumers. Awareness-raising public campaigns, social media campaigns, and childhood education are key elements [114,115]. These communication programs should be tailored to country-specific characteristics such as cultural, political, and economic differences of each society, and should also consider each population’s food-safety knowledge, attitude, and risk perception [116]. Short, clear, and easy-to-remember messages could help consumers balance sustainability and food safety during decision-making about food choice, shopping, food storage, eating out, and management of leftovers.

Food-safety risk communication may be approached through the positive effects of social norms, as with environmental protection issues (for example, failing to sort garbage and recycling and using plastic straws, which are potentially harmful to marine animals, are considered to be careless behaviors by society). Since social norms can effectively influence the relationship between individuals’ risk perception and active food-related behavior [117], establishing positive and correct social norms as standards on a population level could be a primary goal in the communication of food-safety agencies [118]. At the same time, it can be important to focus on emotional motivations as well, for instance, revealing that consumers can make their families and children sick through unsafe practices. For all this to occur, the food-safety problem arising from avoiding food waste or plastic packaging must be made “visible” to consumers. In connection to the cases mentioned in previous sections, such as hyphae of molds and cross-contamination due to improper use of a consumer’s own (textile) bag are typical examples of invisible hazards. Food-waste generation and avoidance of plastic bags are forms of behavior with clearly apparent consequences, while their food-safety aspects are hard to imagine. It should be noted that interventions and risk communication based on social norms can have rebounding outcomes called “the boomerang effect.” This means that the method can successfully transmit the information but its effect on consumer behavior can be the opposite of what is expected. This can be especially true if the target group perceives the communication as oppressive or inconvenient or if the behavior defined in the norm limits their choices [119]. As a result, people might feel discouraged from consumption or even scared of food.

The listed risky behaviors associated with sustainability-related issues mentioned above can assist authorities in the implementation of new risk-communication approaches. While this paper suggests only a few examples of integration of food-safety aspects into emerging sustainability-related market trends, the experience can be generalized to other issues originating from environmentally conscious consumer behavior. The challenges identified may encourage food-safety authorities to rethink risk-communication practices, consider sustainability aspects behind every decision, and boost extensive citizen involvement to tackle these complex issues. Policy-makers should consider consumer behavior and attitudes when issuing new regulations.

As part of new food-safety risk communication policies, a multi-stakeholder approach is needed, based on the understanding that not only the problems are common, but the responsibility and the solution should be also shared. 

## Figures and Tables

**Table 1:** Messages targeting risky behaviors * from the domain of sustainability–safety controversy regarding food waste.

Risky Consumer Behavior in Food-Waste Mitigation	Examples of Seemingly Controversial Food-Safety Advice	Recommendations to Disband Conflicts between Food Safety and Food-Waste Reduction
Consuming food after use-by date based on sensory evaluation	Respect use-by dates [63]. When in doubt, throw it out [64]. Don’t trust your senses [65].	Plan menus ahead of time. Check the labeling during the shopping. Follow the “first in, first out” practice. Differentiate expiration-date types. Keep track of your food stock.
Removing mold from spoiled bread, cheese, jam, rotten vegetables, and fruits and consuming the “clean” parts	Food covered with mold should be thrown away. Don’t risk it; don’t eat it. Discard an entire loaf of bread if it has developed mold [66].	Plan ahead and consider quantities. Check refrigerator temperature. Freeze bread and cheese for elongated shelf life. Know where to store different foods.
Consuming leftovers after storage at inappropriate temperatures for too long and reheating more than once	Leftovers should be stored at 4 °C or below. Never keep leftovers for more than 2–3 days. Reheat leftovers only once [67,68].	Plan portions. Check refrigerator temperature. Split leftover meals into smaller portions. Label dates. Freeze it if you can’t eat it.

* Risky behaviors identified by Skuland et al. (2020) [37].

**Table 2:** Messages targeting risky behaviors from the domain of sustainability–safety controversy regarding the minimizing of plastic packaging.

Risky Consumer Behavior in Minimizing Plastic Packaging	Examples of Seemingly Controversial Food-Safety Advice	Recommendations to Disband Conflicts between Ensuring Food Safety and Minimizing Plastic Packaging
Using a reusable bag many times in a row without washing or sanitizing [106]	Use single-use plastic bags for temporary storage or transportation of RTE (ready-to-eat) food to prevent cross-contamination [37].	Bring your reusable bags to the shop, but pay attention to their proper washing and sanitization (if possible, wash at 60 °C and iron). Wash and sanitize reusable bags dedicated to RTE food after each use. For the industry: Include washing instructions on a label inside each bag.
Using the same reusable bag for RTE and root vegetables [107]	Separate meat, fruits, vegetables, and bakery products during shopping [105]. Pack meat and vegetables in separate bags [37,108].	Dedicate separate reusable bags for bakery products, fruits, and vegetables. Use reusable bags primarily for low-risk food. Use biodegradable and recyclable single-use bags for high-risk food (e.g., raw meat).
Using unlabeled food ingredients during food preparation	Buy food from reliable sources: properly packaged, with proper labeling.	Pay special attention to labeling issues when shopping in a package-free shop: (1) Write the name of the food, date of shopping, expiration date and other possible relevant information onto the container/bag. (2) If there are product tags or printed labels placed next to the product, take one. Apply the FIFO principle in your household. Know where to store different food categories.

## References

[1] Trudel R. (2019). Sustainable consumer behavior. Consum. Psychol. Rev..

[2] Popp J., Oláh J., Kiss A., Temesi Á., Fogarassy C., Lakner Z. (2018). The socio-economic force field of the creation of short food supply chains in Europe. J. Food Nutr. Res..

[3] Civero G., Rusciano V., Scarpato D., Simeone M. (2021). Food: Not Only Safety, but Also Sustainability. The Emerging Trend of New Social Consumers. Sustainability.

[4] Vermeir I., Verbeke W. (2006). Sustainable food consumption: Exploring the consumer “attitude–behavioral intention” gap. J. Agric. Environ..

[5] Veflen N., Scholderer J., Langsrud S. (2020). Situated food safety risk and the influence of social norms. Risk Anal..

[6] Szakos D., Szabó-Bódi B., Kasza G. (2021). Consumer awareness campaign to reduce household food waste based on structural equation behavior modeling in Hungary. Environ. Sci. Pollut. Res..

[7] Yamin P., Fei M., Lahlou S., Levy S. (2019). Using social norms to change behavior and increase sustainability in the real world: A systematic review of the literature. Sustainability.

[8] Cavaliere A., Ventura V. (2018). Mismatch between food sustainability and consumer acceptance toward innovation technologies among Millennial students: The case of Shelf Life Extension. J. Clean. Prod..

[9] Szabó-Bódi B., Kasza G., Szakos D. (2018). Assessment of household food waste in Hungary. Br. Food J..

[10] Seubelt N., Michalke A., Gaugler T. (2022). Influencing factors for sustainable dietary transformation—A case study of German food consumption. Foods.

[11] World Health Organization (2015). Fact Sheet No 399. Food Safety. http://www.who.int/mediacentre/factsheets/fs399/en/.

[12] Guillier L., Duret S., Hoang H.M., Flick D., Nguyen-Thé C., Laguerre O. (2016). Linking food waste prevention, energy consumption and microbial food safety: The next challenge of food policy?. Curr. Opin. Food Sci..

[13] Garske B., Heyl K., Ekardt F., Weber L.M., Gradzka W. (2020). Challenges of food waste governance: An assessment of European legislation on food waste and recommendations for improvement by economic instruments. Land.

[14] World Health Organization Europe (2017). The Burden of Foodborne Diseases in the WHO European Region. https://www.euro.who.int/__data/assets/pdf_file/0005/402989/50607-WHO-Food-Safety-publicationV4_Web.pdf.

[15] Farber J.M., Todd E.C. (2000). Safe Handling of Foods.

[16] World Health Organization (2015). WHO Estimates of the Global Burden of Foodborne Diseases: Foodborne Disease Burden Epidemiology Reference Group 2007–2015.

[17] Centers for Disease Control and Prevention (CDC) (2015). Surveillance for Foodborne Disease Outbreaks, United States, 2015, Annual Report.

[18] Khan I., Miskeen S., Khalil A.T., Phull A.R., Kim S.J., Oh D.H. (2016). Foodborne pathogens: Staphylococcus aureus and Listeria monocytogenes an unsolved problem of the food industry. Pak. J. Nutr..

[19] Kasza G., Szabó-Bódi B., Lakner Z., Izsó T. (2019). Balancing the desire to decrease food waste with requirements of food safety. Trends Food Sci. Technol..

[20] Fava F., Zanaroli G., Vannini L., Guerzoni E., Bordoni A., Viaggi D., Robertson J., Waldron K., Bald C., Esturo A. (2013). New advances in the integrated management of food processing by-products in Europe: Sustainable exploitation of fruit and cereal processing by-products with the production of new food products (NAMASTE EU). New Biotechnol..

[21] Fricz A.S., Ittzés A., Ózsvári L., Szakos D., Kasza G. (2020). Consumer perception of local food products in Hungary. Br. Food J..

[22] Pogutz S., Micale V. (2011). Sustainable consumption and production: An effort to reconcile the determinants of environmental impact. Soc. Econ..

[23] Conrad Z., Niles M.T., Neher D.A., Roy E.D., Tichenor N.E., Jahns L. (2018). Relationship between food waste, diet quality, and environmental sustainability. PLoS ONE.

[24] Scherhaufer S., Moates G., Hartikainen H., Waldron K., Obersteiner G. (2018). Environmental impacts of food waste in Europe. Waste Manag..

[25] Roe B.E., Qi D., Bender K.E. (2020). Some issues in the ethics of food waste. Physiol. Behav..

[26] Welch D., Swaffield J., Evans D. (2018). Who’s responsible for food waste? Consumers, retailers and the food waste discourse coalition in the United Kingdom. J. Consum. Cult..

[27] European Parliament and the Council of the European Union (2018). Directive (EU) 2018/851 of the European Parliament and of the Council of 30 May 2018 amending Directive 2008/98/EC on waste. Off. J. Eur. Union.

[28] National Waste Management Plan Bulgaria. https://projects2014-2020.interregeurope.eu/circpro/news/news-article/12909/national-waste-management-plan-bulgaria/.

[29] Perkoulidis G., Malamakis A., Banias G., Moussiopoulos N. (2022). Development of a Methodological Framework for the Evaluation of the Material and Energy Recovery Potential of Municipal Solid Waste Management: Implementation in Five Greek Regions. Circ. Econ. Environ. Prot..

[30] Hungary Waste Prevention Country Profile 2021. https://www.eea.europa.eu/themes/waste/waste-prevention/countries/hungary-waste-prevention-country-profile-2021/view.

[31] Dobre-Baron O., Nițescu A., Niță D., Mitran C. (2022). Romania’s Perspectives on the Transition to the Circular Economy in an EU Context. Sustainability.

[32] O’Connor C., Gheoldus M., Jan O. (2014). Comparative Study on EU Member States’ Legislation and Practices on Food Donation.

[33] Osaili T.M., Saeed B.Q., Taha S., Omar Adrees A., Hasan F. (2022). Knowledge, Practices, and Risk Perception Associated with Foodborne Illnesses among Females Living in Dubai, United Arab Emirates. Foods.

[34] Food and Agriculture Organization of the United Nations & World Health Organization (2002). Statistical Information on Food-Borne Disease in Europe—Microbiological and Chemical Hazards.

[35] FUSIONS (2016). Estimates for European Food Waste Level. https://www.eufusions.org/phocadownload/Publications/Estimates%20of%20European%20ood%20waste%20levels.pdf.

[36] Redmond E.C., Griffith C.J. (2003). Consumer food handling in the home: A review of food safety studies. J. Food Prot..

[37] Borda D., Didier P., Dumitraşcu L., Ferreira V., Foden M., Langsrud S., Maître I., Martens L., Møretrø T., Nicolau A.I., Skuland S.E. (2020). European Food Safety: Mapping Critical Food Practices and Cultural Differences in France, Norway, Portugal, Romania and the UK..

[38] Watson M., Meah A. (2012). Food, waste and safety: Negotiating conflicting social anxieties into the practices of domestic provisioning. Sociol. Rev..

[39] Tsiros M., Heilman C.M. (2005). The effect of expiration dates and perceived risk on purchasing behavior in grocery store perishable categories. J. Mark..

[40] Kavanaugh M., Quinlan J.J. (2020). Consumer knowledge and behaviors regarding food date labels and food waste. Food Control.

[41] ICF, Anthesis, Brook Lyndhurst, Directorate-General for Health and Food Safety (European Commission), WRAP (2018). Market Study on Date Marking and Other Information Provided on Food Labels and Food Waste Prevention. Final Report. https://op.europa.eu/en/publication-detail/-/publication/e7be006f-0d55-11e8-966a-01aa75ed71a1/language-en.

[42] Abeliotis K., Lasaridi K., Chroni C. (2014). Attitudes and behaviour of Greek households regarding food waste prevention. Waste Manag. Res..

[43] Rawat S. (2015). Food Spoilage: Microorganisms and their prevention. Asian J. Plant Sci..

[44] Junqueira L., Truninger M., Almli V.L., Ferreira V., Maia R., Teixeira P. (2021). Self-reported practices by Portuguese consumers regarding eggs’ safety: An analysis based on critical consumer handling points. Food Control.

[45] Cardoso M.J., Nicolau A.I., Borda D., Nielsen L., Maia R.L., Møretrø T., Ferreira V., Knøchel S., Lngsrud S., Teixeira P. (2021). Salmonella in eggs: From shopping to consumption—A review providing an evidence-based analysis of risk factors. Compr. Rev. Food Sci. Food Saf..

[46] Annunziata A., Agovino M., Ferraro A., Mariani A. (2020). Household Food Waste: A Case Study in Southern Italy. Sustainability.

[47] Olsen M., Gidlund A., Sulyok M. (2017). Experimental mould growth and mycotoxin diffusion in different food items. World Mycotoxin J..

[48] Veflen N., Teixeira P. (2022). Food safety myths consequences for health: A study of reported gastroenteritis incidence and prevalence in UK, Norway and Germany. Food Control.

[49] Matumba L., Monjerezi M., Kankwamba H., Njoroge S.M., Ndilowe P., Kabuli H., Kambewa D., Njapau H. (2016). Knowledge, attitude, and practices concerning presence of molds in foods among members of the general public in Malawi. Mycotoxin Res..

[50] Dantigny P., Conika M., Fontana A., Schorr-Galindo S. (2021). Mycotoxins during Consumer Food Storage. Mycotoxins in Food and Beverages Innovations and Advances Part I.

[51] Rychlik M., Schieberle P. (2011). Model studies on the diffusion behavior of the mycotoxin patulin in apples, tomatoes, and wheat bread. Eur. Food Res. Technol..

[52] Coton M., Dantigny P. (2019). Mycotoxin migration in moldy foods. Curr. Opin. Food Sci..

[53] Skandamis P.N., Yoon Y., Stopforth J.D., Kendall P.A., Sofos J.N. (2008). Heat and acid tolerance of Listeria monocytogenes after exposure to single and multiple sublethal stresses. Food Microbiol..

[54] Juneja V.K., Novak J.S., Eblen B.S., McClane B.A. (2001). Heat resistance of *Clostridium perfringens* vegetative cells as affected by prior heat shock 1. J. Food Saf..

[55] Lorenzo J.M., Munekata P.E., Dominguez R., Pateiro M., Saraiva J.A., Franco D. (2018). Main groups of microorganisms of relevance for food safety and stability: General aspects and overall description. INNOVATIVE Technologies for Food Preservation.

[56] Naranjo M., Denayer S., Botteldoorn N., Delbrassinne L., Veys J., Waegenaere J., Sirtaine N., Driesen R.B., Sipido K.R., Mahillon J. (2011). Sudden death of a young adult associated with *Bacillus cereus* food poisoning. J. Clin. Microbiol..

[57] Hernández-Borges J., Cabrera J.C., Rodríguez-Delgado M.Á., Hernández-Suárez E.M., Saúco V.G. (2009). Analysis of pesticide residues in bananas harvested in the Canary Islands (Spain). Food Chem..

[58] Wang S., Sun H., Liu Y. (2018). Residual behavior and risk assessment of tridemorph in banana conditions. Food Chem..

[59] Mattsson L., Williams H., Berghel J. (2018). Waste of fresh fruit and vegetables at retailers in Sweden–Measuring and calculation of mass, economic cost and climate impact. Resour. Conserv. Recycl..

[60] Redmond E.C., Griffith C.J. (2005). Consumer perceptions of food safety education sources: Implications for effective strategy development. Br. Food J..

[61] Eley C., Lundgren P.T., Kasza G., Truninger M., Brown C., Hugues V.L., Izso T., Teixeira P., Ferré N., Kunszabo A. (2021). Teaching young consumers in Europe: A multicentre qualitative needs assessment with educators on food hygiene and food safety. Perspect. Public Health.

[62] World Health Organization (2018). Risk Communication Applied to Food Safety: Handbook.

[63] NHS England (National Health Services of England) (2020). 10 Ways to Prevent Food Poisoning. https://www.nhs.uk/Livewell/homehygiene/Pages/Foodpoisoningtips.aspx.

[64] University of Wisconsin-Madison Food Safety in Your Pantry: When in Doubt, Throw It Out. https://fyi.extension.wisc.edu/safehealthypantries/step-2-strategies/food-safety-in-your-pantry/when-in-doubt-throw-it-out/.

[65] (2019). SafeConsume Project (H2020 SFS-37 Grant Agreement: 727580). Don’t Trust Your Senses When Comes to Food Safety. https://safeconsume.eu/articles/dont-trust-your-senses-when-comes-to-food-safety.

[66] Food Safety and Inspection Service, U.S. Department of Agriculture (2013). Molds on Food: Are They Dangerous?. https://www.fsis.usda.gov/food-safety/safe-food-handling-and-preparation/food-safety-basics/molds-food-are-they-dangerous.

[67] Government of Canada (2020). Food Safety Tips for Leftovers. https://www.canada.ca/en/health-canada/services/general-food-safety-tips/food-safety-tips-leftovers.html.

[68] U.S. Food and Drug Administration (FDA) (2022). Refrigerator Thermometers—Cold Facts about Food Safety. https://www.fda.gov/food/buy-store-serve-safe-food/refrigerator-thermometers-cold-facts-about-food-safety.

[69] EFSA’s Communication Experts Network (2017). When Food Is Cooking Up a Storm. Proven Recipes for Risk Communications. https://www.efsa.europa.eu/sites/default/files/corporate_publications/files/riskcommguidelines170524.pdf.

[70] Stefan V., van Herpen E., Tudoran A.A., Lähteenmäki L. (2013). Avoiding food waste by Romanian consumers: The importance of planning and shopping routines. Food Qual. Prefer..

[71] Michigan State University (2014). Keep Food Safe by Implementing the “FIFO” System. https://www.canr.msu.edu/news/keep_food_safe_by_implementing_the_fifo_system#:~:text=FIFO%20is%20%E2%80%9Cfirst%20in%20first,and%20use%20them%20more%20efficiently.

[72] European Food Information Council (EUFIC) (2017). Safe Food Storage at Home. https://www.eufic.org/en/food-safety/article/safe-food-storage-at-home.

[73] Wasteless Project (LIFE-FOODWASTEPREV) (2017). How to Store Leftovers. https://maradeknelkul.hu/2017/02/10/a-fott-etelek-tarolasanak-muveszete/.

[74] Verghese K., Lewis H., Lockrey S., Williams H. (2013). The Role of Packaging in Minimising Food Waste in the Supply Chain of the Future.

[75] Estir M., Hasangholipour T., Yazdani H., Nejad H.J., Rayej H. (2010). Food Products Consumer Behaviors: The Role of Packaging Elements. J. Appl. Sci..

[76] Marsh K., Bugusu B. (2007). Food packaging—Roles, materials, and environmental issues. J. Food Sci..

[77] Shah S., Ahmed A., Ahmad N. (2013). Role of packaging in consumer buying behavior. Int. Rev. Basic Appl. Sci..

[78] Martinho G., Pires A., Portela G., Fonseca M. (2015). Factors affecting consumers’ choices concerning sustainable packaging during product purchase and recycling. Resour. Conserv. Recycl..

[79] European Parliament and Council (2015). Directive (EU) 2015/720 of the European Parliament and of the Council of 29 April 2015 amending Directive 94/62/EC as regards reducing the consumption of lightweight plastic carrier bags. Off. J. Eur. Union.

[80] European Parliament and Council (2019). Directive (EU) 2019/904 of the European Parliament and of the Council of 5 June 2019 on the reduction of the impact of certain plastic products on the environment. Off. J. Eur. Union.

[81] European Parliament and Council Directive 94/62/EC of 20 December 1994 on Packaging and Packaging Waste. https://eur-lex.europa.eu/legal-content/EN/TXT/?uri=CELEX%3A01994L0062-20180704.

[82] Schneider D.R., Tomić T., Raal R. (2021). Economic viability of the deposit refund system for beverage packaging waste–identification of economic drivers and system modelling. J. Sust. Dev. Energy Water Environ. Syst..

[83] Diemer A., Nedelciu C.E., Morales M., Batisse C., Cantuarias-Villessuzanne C. (2022). Waste Management and Circular Economy in the French Building and Construction Sector. Front. Sustain..

[84] Assembleia da República (2019). Law n. 76/2019. Diário Repúb..

[85] Assembleia da República (2019). Law n. 77/2019. Diário Repúb..

[86] Dawson L. (2019). ‘Our Waste, our Resources; A Strategy for England’—Switching to a circular economy through the use of extended producer responsibility. Environ. Law Rev..

[87] National Food Chain Safety Office (Nébih) (2021). Results of the 4th Roundtable. https://portal.nebih.gov.hu/documents/10182/1672273/2021_VI_Kerekasztal_Kerdoiv_eredmenyei_Kasza+Gyula.pdf.

[88] Ecoplus, BOKU, Denkstatt, OFI (2020). Food Packaging Sustainability: A Guide for Packaging Manufacturers, Food Processors, Retailers, Political Institutions & NGOs.

[89] Umezuruike L.O. (2013). A review on the role of packaging in securing food system: Adding value to food products and reducing losses and waste. Afr. J. Agric. Res..

[90] Silvenius F., Katajajuuri J.-M., Grönman K., Soukka R., Koivupuro H.-K., Virtanen Y., Finkbeiner M. (2011). Role of Packaging in LCA of Food Products. Towards Life Cycle Sustainability Management.

[91] Obersteiner G., Cociancig M., Luck S., Mayerhofer J. (2021). Impact of Optimized Packaging on Food Waste Prevention Potential among Consumers. Sustainability.

[92] Williams H., Lindström A., Trischler J., Wikström F., Rowe Z. (2020). Avoiding food becoming waste in households—The role of packaging in consumers’ practices across different food categories. J. Clean. Prod..

[93] The Guardian (2015). England’s Shoppers Say Goodbye to Free Plastic Bags. https://www.theguardian.com/environment/2015/oct/05/englands-shoppers-say-goodbye-to-free-plastic-bags.

[94] Hermsdorf D., Rombach M., Bitsch V. (2017). Food waste reduction practices in German food retail. Br. Food J..

[95] European Parliament and the Council (2002). Regulation (EC) No 178/2002 of the European Parliament and of the Council of 28 January 2002 laying down the general principles and requirements of food law, establishing the European Food Safety Authority and laying down procedures in matters of food safety. Off. J. Eur. Union.

[96] Parashar N., Hait S. (2020). Plastics in the time of COVID-19 pandemic: Protector or polluter?. Sci. Total Environ..

[97] Silva A.L.P., Prata J.C., Walker T.R., Campos D., Duarte A.C., Soares A.M., Rocha-Santos T. (2020). Rethinking and optimising plastic waste management under COVID-19 pandemic: Policy solutions based on redesign and reduction of single-use plastics and personal protective equipment. Sci. Total Environ..

[98] Cardoso M.J., Ferreira V., Truninger M., Maia R., Teixeira P. (2020). Cross-contamination events of Campylobacter spp. in domestic kitchens associated with consumer handling practices of raw poultry. Int. J. Food Microbiol..

[99] Kasza G., Csenki E.Z., Izsó T., Scholderer J. (2022). Paradoxical risk mitigation behavior in private households. Food Control.

[100] Agariya A.K., Johari A., Sharma H.K., Chandraul U.N.S., Singh D. (2012). The Role of Packaging in Brand Communication. Int. J. Sci. Eng. Res..

[101] Hall C., Osses F. (2013). A review to inform understanding of the use of food safety messages on food labels. Int. J. Consum. Stud..

[102] Aschemann-Witzel J., De Hooge I., Amani P., Bech-Larsen T., Oostindjer M. (2015). Consumer-related food waste: Causes and potential for action. Sustainability.

[103] Amicarelli V., Bux C. (2020). Food waste measurement toward a fair, healthy and environmental-friendly food system: A critical review. Br. Food J..

[104] Oláh J., Kasza G., Szabó-Bódi B., Szakos D., Popp J., Lakner Z. (2022). Household food waste research: The current state of the art and a guided tour for further development. Front. Environ. Sci..

[105] Matthews C., Moran F., Jaiswal A.K. (2021). A review on European Union’s strategy for plastics in a circular economy and its impact on food safety. J. Clean. Prod..

[106] Barbosa J., Albano H., Silva C.P., Teixeira P. (2019). Microbiological contamination of reusable plastic bags for food transportation. Food Control.

[107] Williams D.L., Gerba C.P., Maxwell S., Sinclair R.G. (2011). Assessment of the potential for cross-contamination of food products by reusable shopping bags. Food Prot. Trends.

[108] Carrasco E., Morales-Rueda A., García-Gimeno R.M. (2012). Cross-contamination and recontamination by Salmonella in foods: A review. Food Res. Int..

[109] Gowman A.C., Picard M.C., Lim L.T., Misra M., Mohanty A.K. (2019). Fruit waste valorization for biodegradable biocomposite applications: A review. BioResources.

[110] Sganzerla W.G., Rosa G.B., Ferreira A.L.A., da Rosa C.G., Beling P.C., Xavier L.O., de Lima Veeck A.P. (2020). Bioactive food packaging based on starch, citric pectin and functionalized with Acca sellowiana waste by-product: Characterization and application in the postharvest conservation of apple. Int. J. Biol. Macromol..

[111] Lahtela V., Silwal S., Kärki T. (2020). Re-Processing of Multilayer Plastic Materials as a Part of the Recycling Process: The Features of Processed Multilayer Materials. Polymer.

[112] Kowalska A., Manning L. (2022). Food Safety Governance and Guardianship: The Role of the Private Sector in Addressing the EU Ethylene Oxide Incident. Foods.

[113] McCarthy B., Liu H.B. (2017). Waste not, want not’: Exploring green consumers’ attitudes towards wasting edible food and actions to tackle food waste. Br. Food J..

[114] Chang H.H., Meyerhoefer C.D. (2021). COVID-19 and the demand for online food shopping services: Empirical Evidence from Taiwan. Am. J. Agric. Econ..

[115] Owusu V., Ma W., Renwick A., Emuah D. (2021). Does the use of climate information contribute to climate change adaptation? Evidence from Ghana. Clim. Dev..

[116] Kasza G., Csenki E., Szakos D., Izsó T. (2022). The evolution of food safety risk communication: Models and trends in the past and the future. Food Control.

[117] Harris K., Depietro R.B., Klein J., Jin D. (2020). The impact of social norms and risk assessment on diners’ reaction to food safety concerns in restaurants. J. Foodserv. Bus. Res..

[118] Scholderer J., Veflen N. (2019). Social norms and risk communication. Trends Food Sci. Technol..

[119] Richter I., Thøgersen J., Klöckner C.A. (2018). A social norms intervention going wrong: Boomerang effects from descriptive norms information. Sustainability.

